# Different rates of endocytic activity and vesicle transport from the apical and synaptic poles of the outer hair cell

**DOI:** 10.1007/s00106-019-0674-y

**Published:** 2019-05-09

**Authors:** C. Harasztosi, A. W. Gummer

**Affiliations:** Section of Physiological Acoustics and Communication, Faculty of Medicine, Eberhard Karls University Tübingen, Elfriede-Aulhorn-Str. 5, 72076 Tübingen, Germany

**Keywords:** Cochlea, Endocytosis, Pinocytosis, Transcytosis, FM dyes, Cochlea, Endozytose, Pinozytose, Transzytose, FM-Farbstoffer

## Abstract

**Background:**

Intense endocytic activity at the apex of outer hair cells (OHCs)—the electromechanical cells of the cochlea—has been demonstrated using the vital plasma-membrane marker FM1-43 and confocal laser-scanning microscopy. Vesicular traffic toward the cell nucleus to distinct locations of the endoplasmic reticulum has also been shown.

**Objective:**

The current study characterizes the dynamics of endocytic activity, as well as apicobasal and basoapical trafficking, using a local perfusion technique that we recently developed and published to visualize bidirectional trafficking in isolated bipolar cells.

**Materials and methods:**

The fluorescent plasma-membrane markers FM1-43 (10 µM) and FM4-64 (10 µM), together with a fluid-phase marker, Lucifer yellow (50 µM), were used to label endocytosed vesicles in isolated OHCs of the guinea pig cochlea. Targets of endocytosed vesicles were examined with a fluorescent marker of subsurface cisternae, DiOC_6_ (0.87 µM). Single- and two-photon confocal laser-scanning microscopy was used to visualize labeled vesicles.

**Results:**

The plasma-membrane markers presented more intense vesicle internalization at the synaptic pole than at the apical pole of the OHC. Intracellular basoapical vesicle trafficking was faster than apicobasal trafficking. Vesicles endocytosed at the synaptic pole were transcytosed to the endoplasmic reticulum system. An intracellular Lucifer yellow signal was not detected.

**Conclusion:**

The larger endocytic fluorescent signals in the synaptic pole and the faster basoapical trafficking imply that membrane internalization and vesicle trafficking are more efficient at the synaptic pole than at the apical pole of the OHC.

## Zusammenfassung

**Hintergrund:**

Intensive endozytische Aktivität am apikalen Pol der äußeren Haarsinneszelle (ÄHZ) – der elektromechanischen Zelle der Cochlea – wurde mit dem vitalen Plasmamembranmarker FM1-43 unter Einsatz konfokaler Laserscanningmikroskopie nachgewiesen. Es wurde gezeigt, dass Vesikel in Richtung Zellkern zu unterschiedlichen Bereichen des endoplasmatischen Retikulums (ER) transportiert werden.

**Fragestellung:**

Ziel der aktuellen Studie war es, die Dynamik der endozytischen Aktivität sowie des apikobasalen und basoapikalen Vesikeltransports mittels einer vor Kurzem von den Autoren entwickelten lokalen Perfusionstechnik zu charakterisieren, welche die Visualisierung von bidirektionalem Vesikeltransport in isolierten bipolaren Zellen ermöglicht.

**Material und Methode:**

Die fluoreszierenden Plasmamembranmarker FM1-43 (10 µM) und FM4-64 (10 µM) sowie der fluoreszierende Fluidphasenmarker Lucifer Yellow (50 µM) wurden verwendet, um endozytierte Vesikel in isolierten ÄHZ der Meerschweinchencochlea zu markieren. Ziele der endozytierten Vesikel wurden mit einem ER-Fluoreszenzmarker, DiOC_6_ (0,87 µM), untersucht. Fluoreszierende Vesikel wurden mittels Einzel- und Zwei‑Photonen-Laserscanningmikroskopie sichtbar gemacht.

**Ergebnisse:**

Die Plasmamembranmarker zeigten eine intensivere Vesikelaufnahme am synaptischen als am apikalen Pol der ÄHZ. Der intrazelluläre Vesikeltransport in basoapikaler Richtung war schneller als in apikobasaler Richtung. Am synaptischen Pol endozytierte Vesikel wurden zum ER transportiert. Ein intrazelluläres fluoreszierendes „Lucifer-Yellow-Signal“ wurde nicht detektiert.

**Schlussfolgerungen:**

Die intensiveren endozytischen Fluoreszenzsignale im synaptischen Pol und der schnellere Transport in die basoapikale Richtung deuten darauf hin, dass Membranaufnahme und Vesikeltransport effizienter am synaptischen als am apikalen Pol der ÄHZ sind.

Hair cells in the sensory epithelium of the cochlea are vulnerable and do not regenerate spontaneously. Therefore, ideally, the cells must function for the entire lifetime of the mammal. However, there is still little known about the membrane- and protein-recycling machinery of hair cells. The present study investigates endocytosis and vesicle trafficking of outer hair cells using fluorescent membrane markers. Understanding of the underlying mechanisms is a prerequisite for the future design of cell-specific medication for hair-cell rehabilitation or regeneration.

## Introduction

Outer hair cells (OHCs)—the electromotile elements of the organ of Corti—are responsible for the high-frequency selectivity as well as low-threshold and broad dynamic range of hearing [2, 6, 20]. In response to a change of transmembrane potential, they produce an electromechanical force of up to at least 50 kHz [10], which acts against frictional forces [8], resulting in the intensity-dependent frequency tuning and amplitude gain of the vibration response of the organ of Corti. The apical pole of the OHC is the site of mechanoelectrical transduction, where hair-bundle deflection causes ion influx accompanied by a change of the transmembrane potential [9]. The basal pole is the place of synaptic communication with nerve fibers from the efferent and afferent systems. The (medial) efferents modulate the electromechanical response of the OHC via calcium-activated changes of the transmembrane potential and cell stiffness [17], which result in a conformation change of the electromotile protein, prestin [35], expressed in high density in the lateral plasma membrane (PM) of the OHC [19], as well as modulation of the electromechanical force produced by the cell. The (type II) afferents are the source of a feedback signal to the soma of the medial efferents in the brainstem [11].

Although cochlear hair cells are functionally vulnerable and not capable of spontaneous regeneration and, hence, required for the entire lifetime of the mammal, there is still little known about the membrane- and protein-recycling machinery driven by endo- and exocytosis. Different types of vesicles have been demonstrated at the apical and basal poles of OHCs [21, 27]. Investigations of the dynamics of membrane recycling using PM fluorescent labeling and confocal microscopy revealed rapid apical endocytic activity and intracellular vesicle trafficking in OHCs [16, 25]. Endocytosis has been proposed to be involved in gentamicin uptake by hair cells and accessory epithelia of the organ of Corti [7]. The development of cell-specific medication to treat sensorineural hearing loss will rely not only on investigations of drug delivery to specific targets within the cochlea [29], but also on an understanding of fundamental cellular internalization processes such as endocytic activity and vesicle trafficking.

Here, we investigate the spatial and temporal properties of endocytic internalization and transport of fluorescent membrane markers applied to OHCs isolated from the guinea pig cochlea. Using a newly developed double-barrel perfusor to apply the markers separately to the basal and apical halves of the OHC [18], we demonstrate greater endocytic uptake in the synaptic region and faster vesicle trafficking in the basoapical direction.

## Methods

### Cell isolation

Outer hair cells were isolated and handled as previously described [18]. Adult pigmented guinea pigs with a weight of 350–900 g (*N* = 21) were used for the study. The data derive from 37 cells isolated from 21 cochleae. Animals were anesthetized by intraperitoneal injection of a mixture of 100 mg/kg ketamine and 4 mg/kg xylazine and were killed by cervical dislocation. The dissected temporal bones were placed in an ice-chilled Hanks’ balanced salt solution (HBSS; Biochrom KG, Berlin, Germany), containing: 137 mM NaCl, 5.4 mM KCl, 1.25 mM CaCl_2_, 4.2 mM NaHCO_3_, 0.81 mM MgSO_4_**∙**7H_2_O, 0.44 mM KH_2_PO_4_, 0.34 mM Na_2_HPO_4_**∙**2H_2_O, 5 mM glucose, and 5 mM HEPES, with an osmolarity of 310 mOsm/l adjusted with D‑(+)-glucose; the pH was 7.3. HBSS was used as the extracellular fluid throughout the preparation steps and experiments. All chemicals were from MERCK (Darmstadt, Germany), unless otherwise stated. Outer hair cells from the apical third of the cochlea were isolated; their lengths were 74 ± 7 µm.

### Perfusion systems

**Fig. 1:**
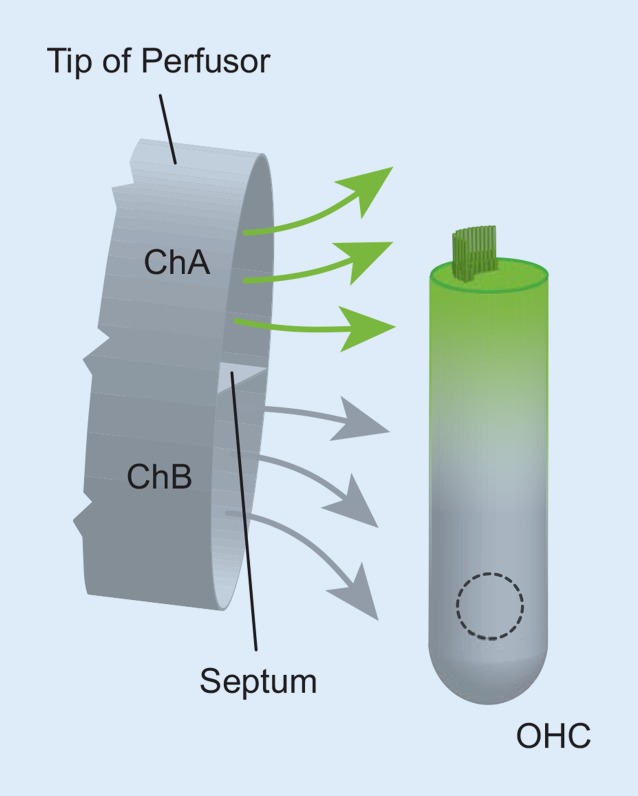
Local perfusion using a double-barrel capillary. The tip of the double-barrel perfusor is positioned close to the basolateral plasma membrane of the isolated outer hair cell (*OHC*). The *septum* separates channel A (*ChA*) and channel B (*ChB*). In this example, HBSS containing green dye is applied from ChA to the apical pole of the cell and dye-free HBSS from ChB to the basal pole. This stimulus configuration allows *apicobasal* vesicle trafficking to be studied; conversely, swapping the channels allows *basoapical* trafficking to be studied. Channels are connected to a peristaltic pump to drive the channels in parallel at a speed that ensures laminar flow around the cell

Single- or double-barrel perfusors (Fig. 1) were used, respectively, to label either both poles or a single pole of the OHC [18]. The tip of the perfusor was positioned near the cell using a micromanipulator (SM5, Luigs and Neumann GmbH, Ratingen, Germany). A peristaltic pump (Ismatec, IDEX Health & Science GmbH, Wertheim, Germany) established laminar flow with a constant flow rate.

Using a single-barrel perfusor enabled homogeneous labeling of the entire PM; the flow rate was 14 µl/min. By contrast, using a double-barrel perfusor enabled labeling of a single pole of the OHC (Fig. 1). The double-barrel perfusor was fabricated from a borosilicate-glass theta capillary with an external diameter of 2 mm (Harvard Apparatus, MA, USA) using a DMZ-Universal Puller (Zeitz Instruments, Augsburg, Germany). To achieve single-pole staining, the perfusor tip was positioned as close as possible to the cell. The coverslip was coated with a cell-and-tissue adhesive (Cell-Tak^TM^) to facilitate cell adhesion. The output flow rate was 3 µl/min per barrel. As recently demonstrated [18], the double-barrel perfusion method is an effective tool for exclusively labeling one pole of bipolar cells such as OHCs.

### Fluorescence microscopy

Imaging was performed using a Zeiss LSM 510 confocal laser-scanning system based on a Zeiss Axioskop2 FS mot microscope (Zeiss, Heidelberg, Germany) equipped with a two-photon laser system (Mira^TM^ 900 Ti:Sapphire Laser pumped by a Verdi V5 Diode-Pumped Laser from Coherent, Santa Clara, USA) and a pinhole diameter of 1 AU. A Zeiss 40 × IR-Achroplan water-immersion objective with NA 0.8 and ZEN2009 software was used.

The fluorescent membrane markers FM1-43 [N-(3-triethylammoniumpropyl)-4-(4-(dibutylamino)styryl)pyridinium dibromide] and FM4-64 [N-(3-triethylammoniumpropyl)-4-(6-(4-(diethylamino)phenyl)hexatrienyl)pyridinium dibromide] were used to visualize endocytosis. FM1-43 is a green-fluorescent, lipophilic styrylpyridinium dye that is commonly used to visualize endocytic and exocytic processes [3], also in cochlear hair cells [15, 16, 18, 23]. Virtually non-fluorescent in aqueous media, the dye rapidly inserts into the PM where it becomes intensely fluorescent; excitation/emission spectral maxima are 473/579 nm, respectively. FM4-64 is a derivative of FM1-43 with similar membrane labeling properties, but exhibits longer wavelength (red) fluorescence making it suitable for double-labeling experiments with lower wavelength dyes [18]; the excitation/emission spectral maxima are 505/725 nm, respectively. Stock solutions of these dyes in a concentration of 10 mM were prepared in DMSO. On the day of the experiment, the dyes were diluted in HBSS to a final concentration of 10 µM.

The fluorescent endoplasmic marker DiOC_6_ (3,3′-dihexyloxacarbocyanine iodide) was used to examine possible targets of endocytosed vesicles in the endoplasmic reticulum (ER). DiOC_6_ is a cell-permeant, green-fluorescent, lipophilic dye, which at low concentrations is selective for mitochondria of live cells and at high concentrations labels other intracellular structures, such as ER [24, 34]. Excitation/emission spectral maxima are 489/506 nm, respectively. For double-labeling experiments with FM4-64 and DiOC_6_, OHCs were first incubated in DiOC_6_ for 1 min, the DiOC_6_ was washed out with extracellular solution, and then the FM4-64 was applied. This FM dye was used instead of FM1-43 because the fluorescence spectra of FM4-64 and DiOC_6_ can be readily separated using bandpass filters. A 1‑mg/ml stock solution of DiOC_6_ was prepared in DMSO. Just before the experiments began, it was diluted with HBSS to a final concentration of 0.87 µM [23].

The fluorescent fluid-phase marker Lucifer yellow (Lucifer yellow carbohydrazide, potassium salt) was used to examine pinocytosis. Lucifer yellow is a well-known dye for studying fluid-phase endocytosis [15]. The dye is hydrophilic, but being anionic cannot permeate the cell membrane by passive diffusion. The potassium-salt of Lucifer yellow has excitation/emission spectral maxima at 428/536 nm, respectively. A 50-mM stock solution of Lucifer yellow was prepared in DMSO. On the day of the experiments, it was diluted with HBSS to a final concentration of 50 µM. This concentration was chosen to be of the same order of magnitude as that of the FM dyes (10 µM).

FM and DiOC_6_ were excited with an argon laser with a wavelength of 488 nm and the emitted light was collected with bandpass filters: 505–750 nm for FM1-43, 650–710 nm for FM4-64, and 500–550 nm for DiOC_6_. For the double-labeling experiments with FM4-64 and DiOC_6_, the emitted light was separated into two channels using a dichroic beam splitter (NFT545). Lucifer yellow was excited by the two-photon laser (840 nm) and the emission recorded below 650 nm using a short-pass filter (KP650).

Except for the Lucifer yellow experiments, fluorescence signals were corrected offline for the average background fluorescence level, which was measured extracellularly just before the start of dye application in a region free of cell remnants.

All dyes were purchased from Thermo Fisher Scientific, Inc. (Waltham, MA, USA).

### Data analysis

The detection threshold, used for calculating signal delay, was set to 20% of the saturation intensity from the PM region of interest (ROI) near the cell pole where the dye was applied. Linear regression, with software in Origin (Ver. 7, OriginLab Corporation, Northampton, MA, USA), was used to estimate the time at which the signal reached/crossed the 20% value. Linear regression was also used for calculating the mean speed of vesicle trafficking. Average data are presented as mean and standard deviation. Statistical tests were performed with the Student’s *t* test and considered significant at *p* < 0.05. Test results are given as *p* and *t*_f_ values, where *f* denotes the degrees of freedom.

## Results

### Apicobasal and basoapical trafficking

**Fig. 2:**
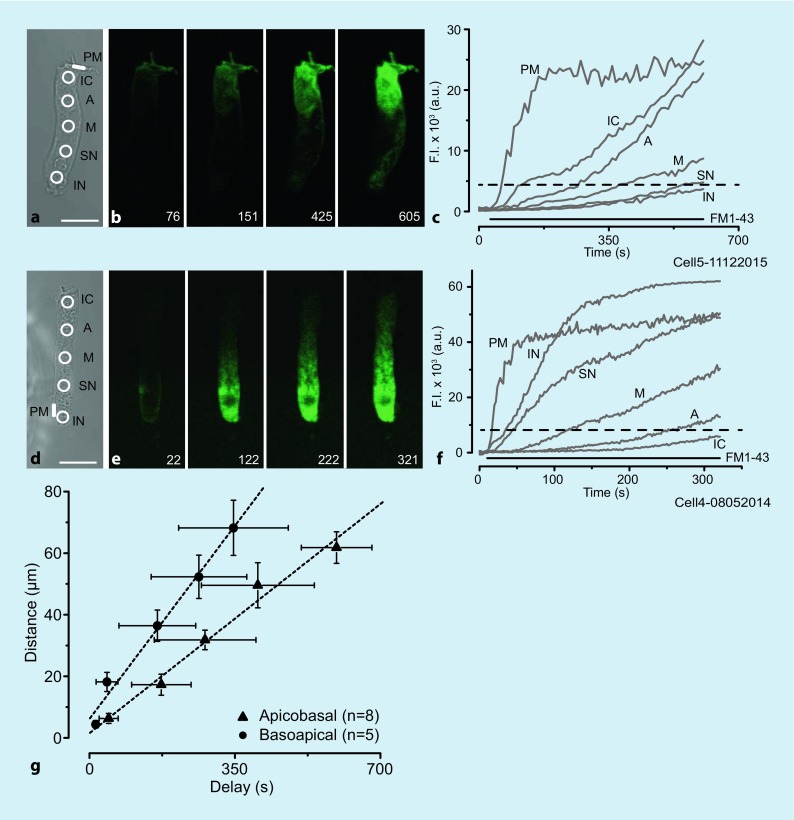
Uptake and trafficking of FM1-43 applied to the apical or basal region of the isolated OHC. **a**–**c** Apical labeling and apicobasal trafficking. **d**–**f** Basal labeling and basoapical trafficking. Data are from different cells. **a**, **d** Phase-contrast images showing the regions of interest (ROIs) used for analysis (*circles* and the *rectangle)*: *IC* infracuticular, *A* apical, *M* middle, *SN* supranuclear, *IN* infranuclear, *PM* plasma membrane. *Scale bar*: 20 µm. The tip of the double-barrel capillary is positioned near the cell from the left side (not shown). **b**, **e** Fluorescence images showing the spatial and temporal courses of cell labeling. *Numbers* in the *bottom right corners* indicate experimental time in seconds. **c**, **f** Fluorescence signal intensities calculated from the ROIs in panel **a** and **d**, respectively. *Dashed black line*: 20% of the steady-state signal level in the PM ROI, used to estimate the signal delay to a given ROI relative to the PM ROI. *Horizontal black line:* FM1-43 application period. **g** Average distance of ROIs from a cell pole as function of time delays of the ROIs relative to the PM ROI for *apicobasal* (8 cells) and *basoapical* (5 cells) vesicle traffic. *Error bars*: standard deviations. *Dashed lines*: linear regression lines used to estimate trafficking speed

Endocytic activity was investigated separately at the apical and basal poles of the isolated OHC (Fig. 2). In the first example shown (Fig. 2a–c), the PM marker FM1-43 was applied to the apical pole of the OHC using the double-barrel perfusor (Fig. 2a). Channel A (ChA) contained 10 µM FM1-43 in HBSS while channel B (ChB) contained dye-free HBSS to prevent apical pole labeling. The spatial distribution and the temporal course of the intracellular dye (Fig. 2b) imply that FM-labeled vesicles traffic toward the base of the cell. Fig. 2c shows the time course of the fluorescence signal intensities in the ROIs indicated in Fig. 2a. The dashed line denotes 20% of the saturation intensity from the apically located PM ROI. This 20% value, arbitrarily set as a threshold signal value, was used to calculate the signal delays to the ROIs relative to the PM ROI. Distances of the ROIs from the apical pole are plotted as a function of signal delay in Fig. 2g (*triangles*) for the average of eight cells. Based on linear regression, the slope yields an average apicobasal speed of 0.11 ± 0.01 µm/s. The distance–axis intercept locates the PM ROI, on average, at 1.52 ± 3.09 µm from the apical pole, which is not significantly different from 0 µm (*t*_7_ = 1.39, *p* = 0.21).

The same protocol was used to investigate basoapical trafficking, the FM1-43 dye being applied to the basal half of the cell (ChB) and the dye-free HBSS to the apical half (ChA) to prevent access of dye to that region (Fig. 2d–f). Signal delay as a function of distance from the basal pole is shown in Fig. 2g (*circles*) for five cells. The average apicobasal speed is 0.18 ± 0.01 µm/s. The distance–axis intercept locates the PM ROI, on average, at 6.25 ± 2.73 µm from the basal pole. In other words, the basoapical speed is significantly greater than the apicobasal speed (*t*_6_ = 12.17, *p* = 9.37.10^−6^), indicating more intense trafficking toward the apex of the cell.

For the apical application, the florescent signal in the infracuticular ROI required, on average, 45.1 ± 22.8 s (*N* = 8) to reach the 20% threshold, whereas for the basal application, on average, only 13.6 ± 6.4 s (*N* = 5) was required for the infranuclear ROI, relative to the respective PM ROIs at or near the poles. The significantly smaller delay for the infranuclear ROI (*t*_11_ = 3.44, *p* = 0.003) indicates that vesicle formation in the synaptic pole is more intense than that at the apex of the OHC.

### Targets of basal endocytosed vesicles

**Fig. 3:**
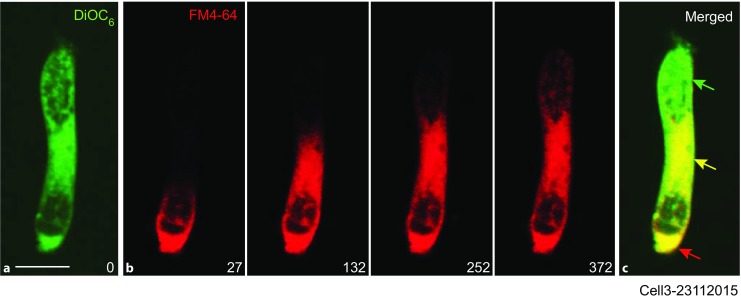
Targets of basal endocytosed vesicles. **a** Endoplasmic reticulum of an outer hair cell labeled with DiOC_6_ (0.87 µM). The image was taken after a 1-min incubation in the dye. *Scale bar*: 20 µM. **b** Spatial and temporal courses of fluorescent vesicles of FM4-64 (10 µM) for dye application at the basal pole. *Numbers* in the *bottom right corners* indicate experimental time in seconds. **c** Image of panel **a** merged with the last image in panel **b**, the yellow staining demonstrating colocalization of DiOC_6_ and FM4-64 labeling

Until now, targets of vesicles formed at the synaptic pole of OHCs had not been demonstrated. Therefore, here, the endoplasmic reticulum of OHCs was labeled with DiOC_6_ (Fig. 3a, *green*) and compared with vesicle targets of the fluorescence PM marker FM4-64 applied to the basal pole (Fig. 3b, *red*). The yellow signal in Fig. 3c indicates colocalization of DiOC_6_ and FM4-64. Similar observations were made in a total of 14 cells. These observations imply that basally endocytosed vesicles are transcytosed to the endoplasmic reticulum.

### Molecular weight limit of endocytosed molecules

**Fig. 4:**
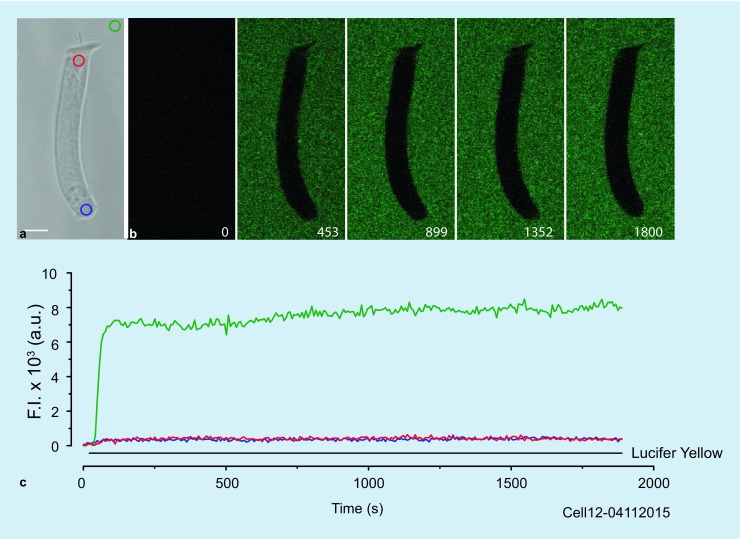
Molecular weight limit for uptake by pinocytosis. **a** Phase-contrast image showing intracellular (*red and blue circles*) and extracellular (*green circle*) positioned ROIs. Scale bar: 10 µm. **b** Fluorescence images showing Lucifer yellow labeling of extracellular fluid only. *Numbers* in the *bottom right corners* indicate experimental time in seconds. **c** Fluorescence signal intensities for the ROIs in panel **a**. *Horizontal black line*: Lucifer yellow application period

To investigate the maximum possible molecular weight (MW) for a substance to be internalized by fluid-phase endocytosis (pinocytosis), Lucifer yellow (MW = 522 Da) was applied to the OHC and the development of intracellular fluorescence recorded (Fig. 4). In these experiments (*N* = 10), a single-barrel perfusor was used to apply the stain homogeneously around the entire cell. To avoid recording of scattered emitted light from extracellular dye, fluorescence was induced and recorded with two-photon confocal microscopy. Despite the presence of a constant and high-intensity extracellular signal and an extensive application time of the dye (>30 min), an intracellular fluorescence signal was not detected above the background noise level (Fig. 4). Importantly, the displayed intracellular signals represent noise derived from the extracellular signal because the intracellular signal dropped to zero when the Lucifer yellow application was terminated and extracellular solution applied (not illustrated). In the absence of a detectability problem, this result implies that anionic molecules with an MW greater than 500 Da cannot be internalized by pinocytosis.

## Discussion

Different types and sizes of vesicles have been demonstrated at the infracuticular [21] and synaptic [33] pole of hair cells using electron microscopy and horseradish peroxidase labeling, respectively. Investigations using the PM marker FM1-43 show that vesicles formed from the reticular lamina transcytose to distinct intracellular compartments that are part of the elaborate endoplasmic reticulum system of the OHC [16, 23]. For isolated bipolar cells such as the OHC, we developed a double-barrel perfusion system to enable the investigation of endocytosis and transcytosis separately from either the apical or the basal pole [18]. Using a pinocytosis and phagocytosis inhibitor, phenylarsine, it was unequivocally demonstrated that the dye uptake is an endocytic process [18]. Furthermore, by incubating the cell in an inhibitor of the motor molecule kinesin, monastrol, we showed that basoapical transcytosis is partially due to a kinesin trafficking mechanism [18].

**Fig. 5:**
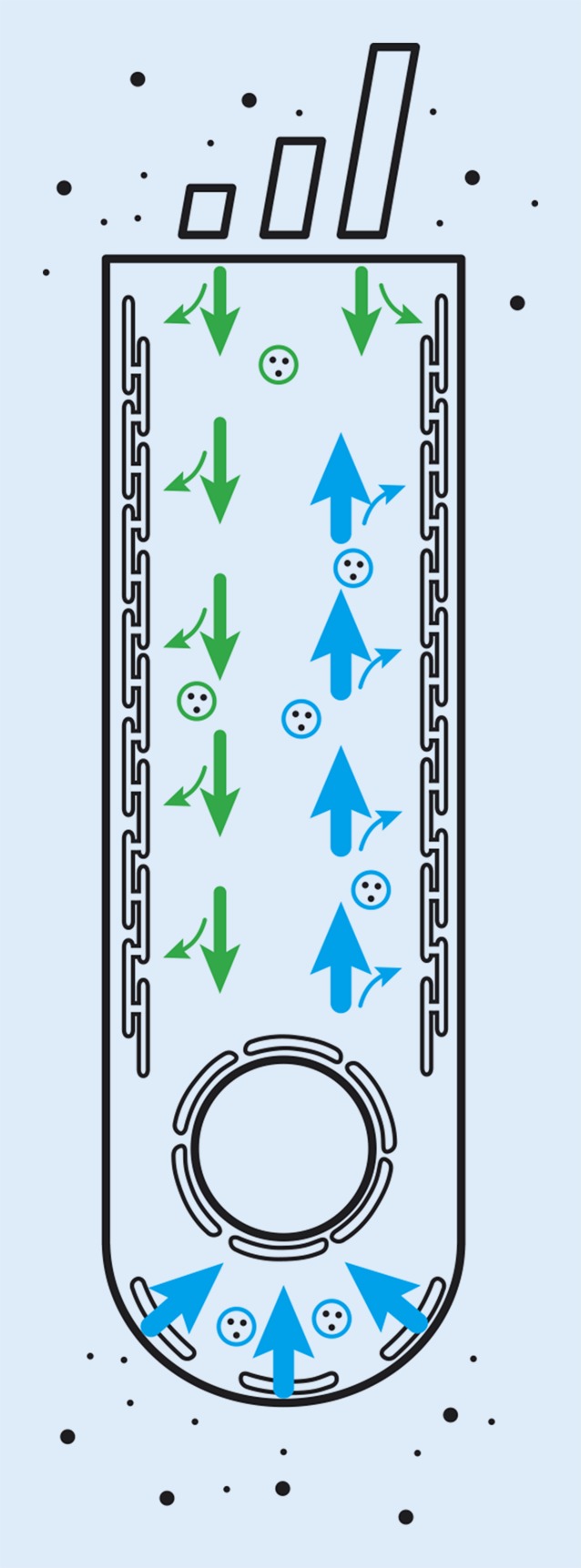
Graphical summary. Endocytic activity of the outer hair cell is more pronounced at the synaptic pole and vesicle trafficking is faster toward the apex

Here, using the double-barrel perfusor, it was found that endocytosis at the synaptic pole and basoapical trafficking are more intense than endocytosis at the apical pole and apicobasal trafficking. Double-labeling experiments with DiOC_6_ and FM4-64 imply that vesicles internalized at the synaptic pole traffic to the endoplasmic reticulum. The findings are summarized graphically in Fig. 5.

Experiments with the low-molecular-weight, fluid-phase endocytic, fluorescent marker Lucifer yellow indicated that pinocytic internalization of anionic molecules is limited to MWs of no more than 500 Da. However, the Lucifer yellow experiments are considered preliminary because if, indeed, there were pinocytic uptake, the number of vesicles might have been insufficient to produce a detectable signal. In these experiments, we limited the concentration of Lucifer yellow (50 µM) to be similar to that used for the PM endocytosis markers (10 µM). Griesinger et al. (2002) demonstrated pinocytic uptake of Lucifer yellow by inner hair cells for high concentrations (20 mM) and long incubation times (60 min). Future MW experiments should examine the pinocytic uptake of fluorescent markers of much greater extinction coefficient and a higher quantum yield than for Lucifer yellow.

### Possible routes of FM1-43 entry into hair cells

It has been shown that FM1-43 can penetrate mechanoelectrical transducer (MET) channels in hair cells of immature cochlea cultures [12], hair cells of embryonic mice [13], developing hair and sensory cells [26], cultured chick auditory papilla hair cells [32], cultured zebrafish larvae lateral line organs [31], and Xenopus larvae lateral line organs [28]. In addition, Crumling et al. [5] used hair cells of chicks 5–10 days post-hatch and showed PPADS- and suramin-dependent AM1-43 entry, implying that FM dyes can also penetrate P2X receptors. To avoid this type of nonspecific labeling, recently two techniques were developed: (1) photo-oxidation electron microscopy [22], whereby the FM dye taken up by the organelles is converted to a dark precipitate visible electron microscopically, and (2) instead of using an FM dye, a novel endocytotic probe impermeable to MET-channels, called membrane-binding fluorophore-cysteine-lysine-palmitoyl group, was used [30]. For both techniques, experiments with IHCs from mice at postnatal day 14–18 yield vastly different patterns of endocytotic signals compared with those found for when uptake is also through the MET channels. Therefore, there is still ongoing controversy about whether FM1-43 does indeed reliably report endocytosis in (P14–18) mice.

However, for the mature cochlea of the guinea pig, there is no evidence of FM-1-43 uptake through the MET channels or P2X receptors. For the inner hair cell (IHC), it has been demonstrated, using an in-situ cochlea preparation from young adult guinea pigs, that neither the MET-channel blockers dihydrostreptomycin or D‑tubocurarine nor the lesioning of tip links with BAPTA treatment influences FM1-43 labeling [15]. Similar results were reported for OHCs in situ using the same animal and preparation as in the IHC study; namely, that FM1-43 labeling was not influenced by these MET-channel blockers or BAPTA [16]. By applying FM1-43 to isolated OHCs from the mature, pigmented guinea pig cochlea, we also demonstrated that block of MET channels by dihydrostreptomycin or of P2X receptors by PPADS had no influence on the FM1-43 labeling [23]. Therefore, for the experimental model on which the present results are based, there is no evidence of FM1-43 entering through MET channels or P2X receptors of hair cells of the functionally mature cochlea. In other words, the available evidence convincingly suggests that in the present experiments FM1-43 uptake was via endocytic activity.

### Physiological relevance of endocytosis in OHCs

Plasma-membrane Ca^2+^ ATPase (PMCA) activity is crucial for removing Ca^2+^ from the cytosol of the stereocilia and, therefore, for protecting hair cells from Ca^2+^ overload [4]. It has been proposed that apical endocytic activity of hair cells is involved in recycling of PMCA molecules of the hair bundle [14].

Receptors and ion channels of the efferents and afferents located at the synaptic pole of the OHC may require continuous replacement. However, the colocalization data with DiOC_6_ and FM4-64 show that vesicles formed at the synaptic pole of OHCs traffic basoapically, targeting the endoplasmic reticulum. Therefore, in addition to PM recycling, proteins such as acetylcholine receptors might also be involved.

It has been proposed that aminoglycoside uptake might be regulated by endocytosis [7]. Although it has been suggested that aminoglycosides enter hair cells through transducer channels in cultured organs from newborn rats [1], the significance of endocytosis-dependent aminoglycoside uptake at the synaptic pole cannot be excluded because aminoglycosides such as gentamicin have a relatively low MW (450–478 Da), which is below the MW limit suggested in this study for pinocytic internalization. With the aid of the double-barrel perfusor, experiments with fluorescent-labeled gentamicin could address the possibility of its endocytic internalization.

## Practical conclusion

- Fluorescent membrane markers enable the investigation of the dynamics of endocytic activity and vesicle trafficking in outer hair cells.
- Such investigations are essential for characterizing cellular mechanisms required not only for membrane and for protein recycling but also for intra- and intercellular communication.
- Mechanisms of vesicle formation and the cargo-specific targets are still debated for outer hair cells.
- Using a recently designed local-perfusion system, the current study demonstrates that basal endocytosis and basoapical trafficking are more intense than apical endocytosis and apicobasal trafficking.
- When coupled with molecular and pharmacological investigations of uptake and trafficking mechanisms, this finding should offer insight into underlying cellular mechanisms, which is a prerequisite for designing cell-specific medication for hair cells in the future.

## Abbreviations

A: Apical
AU: Airy unit
a.u.: Arbitrary units
ChA: Channel A
ChB: Channel B
Da: Dalton
DiOC_6_: 3,3′-Dihexyloxacarbocyanine iodide
DMSO: Dimethyl sulfoxide
FM1-43: N-(3-Triethylammoniumpropyl)-4-(4-(dibutylamino)styryl)pyridinium dibromide
FM4-64: N-(3-Triethylammoniumpropyl)-4-(6-(4-(diethylamino)phenyl)hexatrienyl)pyridinium dibromide
HBSS: Hanks’ balanced salt solution
HEPES: N-2-Hydroxyethylpiperazine-N′-2-ethanesulfonic acid
IC: Infracuticular
IHC: Inner hair cell
IN: Infranuclear
Lucifer yellow: Dipotassium salt of Lucifer yellow carbohydrazide
M: Middle
MET: Mechanoelectrical transducer
MW: Molecular weight
OHC: Outer hair cell
PM: Plasma membrane
PPADS: Pyridoxal phosphate-6-azo(benzene-2,4-disulfonic acid)
ROI: Region of interest
SN: Supranuclear
